# Palladium-catalyzed Sonogashira coupling reactions in γ-valerolactone-based ionic liquids

**DOI:** 10.3762/bjoc.15.284

**Published:** 2019-12-03

**Authors:** László Orha, József M Tukacs, László Kollár, László T Mika

**Affiliations:** 1 Department of Chemical and Environmental Process Engineering, Budapest University of Technology and Economics Műegyetem rkp. 3, Budapest, H-1111, Hungary; 2 Institute of Isotopes, Konkoly-Thege Miklós str. 29-33. Budapest, H-1121, Hungary; 3 Department of Inorganic Chemistry, University of Pécs and János Szentágothai Research Center and MTA-PTE Research Group for Selective Chemical Syntheses, Ifjúság u. 6.; Pécs, H-7624, Hungary

**Keywords:** catalysis, cross coupling, green chemistry, ionic liquids

## Abstract

It was demonstrated that the γ-valerolactone-based ionic liquid, tetrabutylphosphonium 4-ethoxyvalerate as a partially bio-based solvent can be utilized as alternative reaction medium for copper- and auxiliary base-free Pd-catalyzed Sonogashira coupling reactions of aryl iodides and functionalized acetylenes under mild conditions. Twenty-two cross-coupling products were isolated with good to excellent yields (72–99%) and purity (>98%). These results represent an example which proves that biomass-derived safer solvents can be utilized efficiently in common, industrially important transformations exhibiting higher chemical and environmental efficiency.

## Introduction

In the past few decades, the transition-metal-catalyzed coupling reaction has represented one of the most powerful and atom economical strategies for the efficient assembly of new carbon–carbon bonds. It has therefore become the most attractive approach to the synthesis of a wide range of functionalized organic molecules from laboratory to industrial scale [1–3]. Among these tools, the Pd-catalyzed coupling reactions have received substantial attention, due to the mild operation conditions, excellent functional group tolerance and chemoselectivity as well as wide applicability from syntheses of common building blocks to agrochemicals, just to name a few advantages [4–6]. From the series of palladium-assisted C–C bond formation, the Sonogashira coupling reaction has been identified as a viable synthetic method for the preparation of various alkenyl- and arylacetylenes [7–8] having great importance in organic synthetic schemes of the pharmaceutical industry.

From the environmental point of view, the Sonogashira reactions are usually performed in fossil-based common organic reaction media having high vapor pressure even at higher temperatures, toxicity, flammability, etc., which could result in several serious environmental concerns, especially when they are released into the atmosphere. According to the FDA guidelines [9], the typical solvents of Sonogashira reactions such as toluene [10], THF [11], DMF [12], NMP [13], DMA [14], or MeCN [15] are classified into Class 2, of which applications should be strictly limited, particularly in pharmaceutical industry. To develop an environmentally benign alternative of this useful method, the reaction has been extended to green solvents such as water [16], fluorous solvents [17], supercritical CO_2_ [18], and very recently γ-valerolactone [19]. The series of these alternative media can implicitly be continued by ionic liquids (ILs) [20], which have attracted considerable attention, due to their extremely low vapor pressure, good solvating properties, reasonable thermal stability, and easily tuneable physical properties [21]. Accordingly, the Sonogashira reactions were also successfully performed in conventional ionic liquids such as [BMIM][PF_6_] [22–25], [BMIM][BF_4_] [23], [HMIM][BF_4_] [24], [EMIM][NTf_2_] [26], [nBuPy][X] (X = PF_6_^–^, BF_4_^–^, NO_3_^–^) [27], [DectBu_3_P][BF_4_] [28]. It was found that some of these systems could operate copper-free [25,27,29] and/or auxiliary base-free conditions [30]. Recently, some “designer" ionic liquids were also developed for this purpose [29–33] from which an imidazolium-based piperidine-appended one could act as task specific compound operating either as a solvent in itself [31] or as an additive to the common ionic liquids [30,33]. It should be noted; however, significant catalyst loadings (5–10 mol %) were necessary to obtain reasonable product yields for latter reactions.

Although the Sonogashira coupling is a well-studied transformation, it has not been carried out in γ-valerolactone-originated ILs, which can act as solvent, ligand and base. Thus, the preparation of various acetylenes in a partially or even biomass-based solvent without any auxiliary material could further control and reduce the environmental impacts of this industrially important transformation.

Herein we report a study on the palladium-catalyzed copper- and added base-free Sonogashira coupling reactions to synthesize various acetylenes in γ-valerolactone-based ionic liquids under mild conditions.

## Results and Discussion

**Scheme 1:**
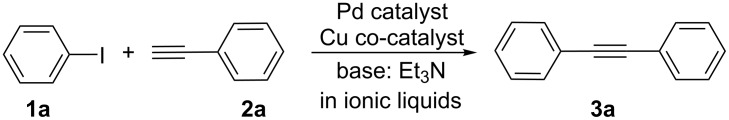
Palladium-catalyzed Sonogashira cross-coupling of iodobenzene (**1a**) and phenylacetylene (**2a**) in ionic liquids.

We recently demonstrated that copper-catalyzed Ullmann-type N–C coupling reactions could be performed in tetrabutylphosphonium 4-alkoxyvalerate-type ionic liquids, which can easily be synthesized from the renewable platform chemical γ-valerolactone and have negligible vapor pressures even at high temperatures [34]. In order to extend the applicability of valerate-based ionic liquids, the conventional imidazolium-type media and tetrabutylphosphonium 4-ethoxyvalerate ([TBP][4EtOV]) were compared in the coupling of iodobenzene (**1a**) and phenylacetylene (**2a**) as a model reaction (Scheme 1) under typically used “Sonogashira conditions” [7,35].

Because the role of ionic liquids as coordination agents for transition metal species was demonstrated [36–37] and palladium carboxylate complexes are well-known compounds, it can be proposed that the carboxylate group of the 4-ethoxyvalerate anion could stabilize the catalytically active species and therefore the ligand can be eliminated from the catalytic system.

Complete conversions of **1a** to diphenylacetylene (**3a**) were detected in the presence of Pd, Cu, and triethylamine (Et_3_N) in all the ILs (Table 1, #1). It should be noted that the typically utilized base, Et_3_N has a foul smell and could affect the product isolation procedure. In addition, it is well-established that the presence of Cu(I) salt can promote the in situ formation of some Cu(I) acetylides, which can readily undergo oxidative homocoupling of alkynes even in their slight excess in the reaction mixture [15,38–39]. Thus, the elimination of these auxiliary materials could result in an environmental benign alternative protocol. Hence, the scope of the reaction was investigated with a combination of palladium, copper co-catalyst and Et_3_N as a base. By elimination of toxic Et_3_N (LD_50(rat, oral)_ = 560 mg/kg) [40], no reactions were detected in imidazolium-type ILs. However, complete conversion of **1a** was observed in [TBP][4EtOV], without Et_3_N, proving that the solvent can act as a base in itself (Table 1, #2) as it was demonstrated for Cu-catalyzed C–N coupling reactions [34].

**Table 1:** Sonogashira coupling reaction of iodobenzene (**1a**) and phenylacetylene (**2a**) in different ionic liquids.^a^

Conversion of iodobenzene in the presence of			
Ionic liquid	Pd, Cu, and Et_3_N (#1)	Pd and Cu (#2)	Pd (#3)

[BMIM][BF_4_]	>99%	<1%	<1%
[EMIM][BF_4_]	>99%	<1%	<1%
[BMIM][Octyls]^b^	>99%	<1%	<1%
[BMIM][PF_6_]	>99%	<1%	<1%
[TBP][4EtOV]	>99%	>99%	>99%

^a^Reaction conditions: 0.8 mL ionic liquid, 0.5 mol % PdCl_2_(PPh_3_)_2_, 1 mol % CuI, 0.75 mmol phenylacetylene, 0.5 mmol iodobenzene, 0.75 mmol Et_3_N, *T* = 55 °C, *t* = 3 h. ^b^Octyls: octylsulfate anion.

When, we attempted to couple **1a** and **2a** in the absence of any copper salt as cocatalyst, complete formation of **3a** was also detected after 3 h revealing that copper can be eliminated from the system without any decrease in the system’s efficiency.

**Figure 1:**
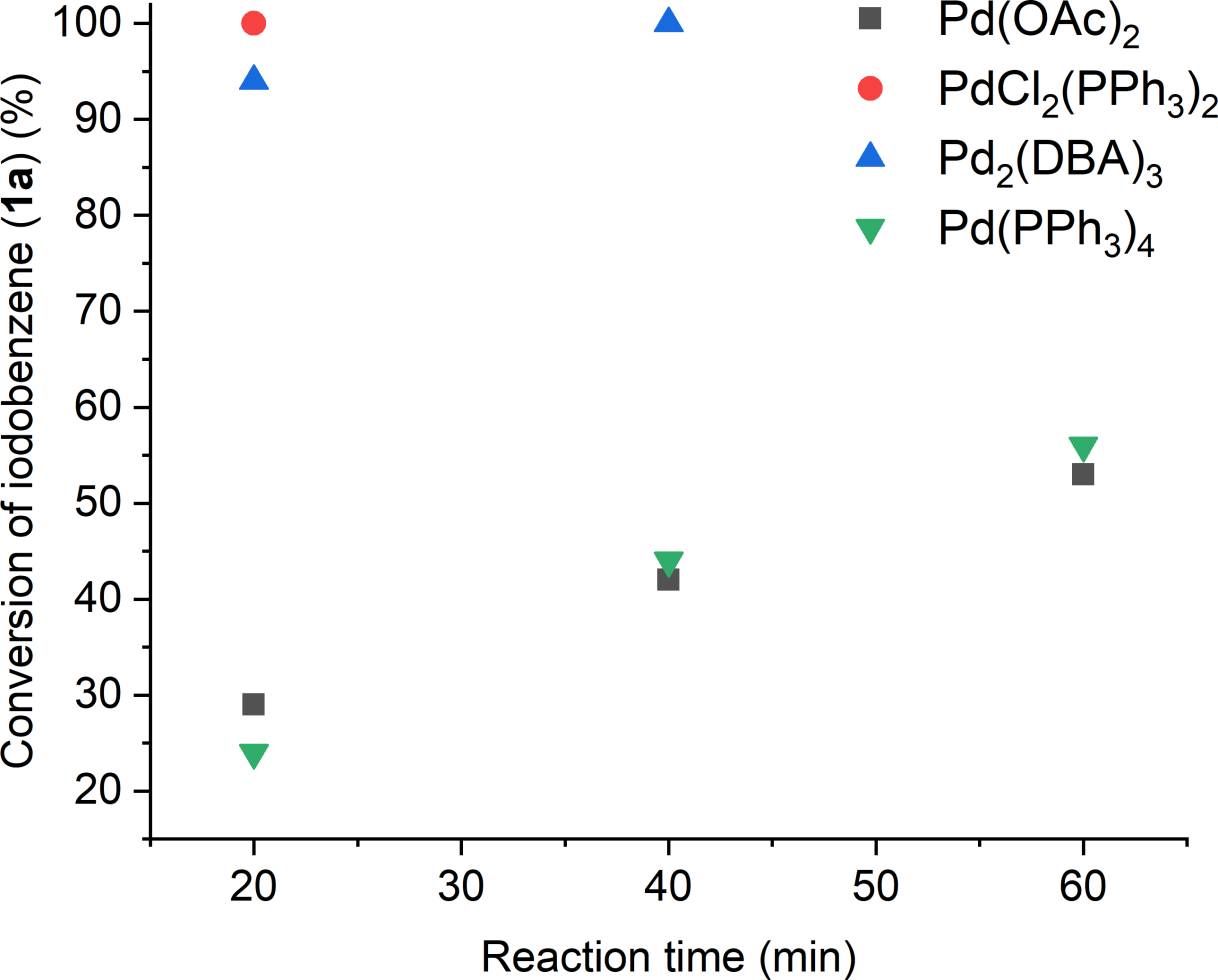
Effect of catalyst precursors used in Sonogashira coupling reaction of iodobenzene (**1a**, 0.5 mmol) and phenylacetylene (**2a**, 0.75 mmol). Reaction conditions: 0.8 mL [TBP][4EtOV], 0.5 mol % catalyst, *T* = 55 °C.

The source of palladium could also have a significant effect on the reaction’s performance [23]. It was found that while Pd(PPh_3_)_4_, palladium acetate (Pd(OAc)_2_), PdCl_2_(PPh_3_)_2_, and tris(dibenzylideneacetone)dipalladium (Pd_2_(DBA)_3_) all catalyzed the Sonogashira reaction, the PdCl_2_(PPh_3_)_2_ precursor turned out to have the best activity in the light of reaction rates (Figure 1). In the presence of 0.5 mol % of PdCl_2_(PPh_3_)_2_ the treatment of 0.5 mmol **1a** with 0.75 mmol of **2a** afforded complete conversion of **1a** in 40 min. Similar performance of PdCl_2_(PPh_3_)_2_ were reported by Ryu, when [BMIM][PF_6_] was used as reaction medium [26]; however, by the use of 10 times higher catalyst loading.

The moisture content of the reaction environment could have a significant effect on the efficiency of a transition-metal-catalyzed reaction. Because [TBP][4EtOV] was isolated from an aqueous solution, the investigation of possible influence of the residual water content on the reaction was highly desired. We found that no decrease in the formation of **3a** was detected when the moisture content was varied between 0.05 and 5.0 wt % (Table 2). Since the method is hardly sensitive to the residual moisture content, to exclude water from the reaction mixture no special pretreatment or handling of the solvent is necessary.

**Table 2:** Effect of water content on Sonogashira coupling of iodobenzene (**1a**) and phenylacetylene (**2a**).^a^

Entry	Water content (wt %)	Isolated yields of **3a** (%)

1	0.05	85
2	1.0	86
2	2.5	82
4	5.0	83

^a^Reaction conditions: 0.8 mL [TBP][4EtOV], 0.5 mol % PdCl_2_(PPh_3_)_2_, 0.75 mmol phenylacetylene, 0.5 mmol iodobenzene, *T* = 55 °C, *t* = 3 h.

Hereafter, the air-stable and readily available PdCl_2_(PPh_3_)_2_ was selected as a catalyst precursor to facilitate C–C bond couplings involving various iodoaromatic compounds (**1a**–**l**) and phenylacetylene (**2a**) in [TBP][4EtOV] in the absence of any additional ligands and auxiliary base at 55 °C for 3 h. In general, the catalytic system could be applied to various iodoarene substrates and the substrate reactivity was not influenced dramatically by the electronic parameters of the substituents. Both electron-withdrawing (chloro, fluoro and bromo) and electron-donating (methyl, methoxy) groups were tolerated on the aryl iodide (Table 3, entries 2–7). Under identical conditions, 2-iodothiophene, and iodopyridine derivatives could also easily be converted to the corresponding acetylene with good or even excellent isolated yields (**3i**–**n**). When 2-amino-3-iodopyridine (**1i**) was converted no C–N bond formation was detected excluding the copper impurities assisted Ullmann-coupling reactions. When 2-chloro-5-iodopyridine (**1l**) was reacted with 1 equiv of **2**, 2-chloro-5-(2-phenylethynyl)pyridine (**3l**) was isolated as product with a yield of 72%. By the use of 2.5 equiv of **2**, the chloro group also undergoes a coupling reaction to form 2,5-bis(2-phenylethynyl)pyridine (**3m**) under identical conditions (Table 3, entries 12 and 13).

**Table 3:** Palladium-catalyzed Sonogashira coupling of various iodoaromatic compounds (**1a–l**) with phenylacetylene (**2a**).^a^

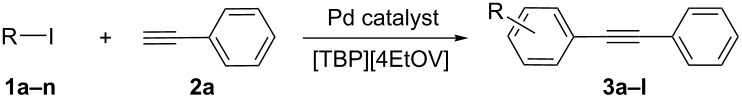					

#	Iodoaromatic compounds		Product		Yield (%)^b^

1	**1a**	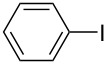	**3a**		85
2	**1b**	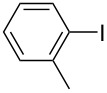	**3b**		96
3	**1c**	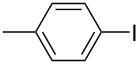	**3c**		95
4	**1d**	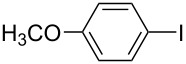	**3d**		82
5	**1e**	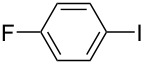	**3e**		80
6	**1f**	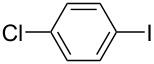	**3f**		87
7	**1g**	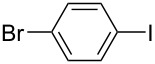	**3g**		52
8	**1h**	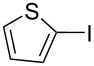	**3h**		80
9	**1i**	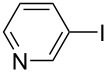	**3i**		75
10	**1j**	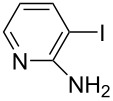	**3j**		93
11	**1k**	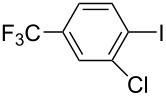	**3k**		79
12	**1l**	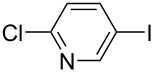	**3l**		72
13^c^	**1l**	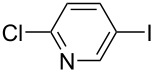	**3m**		69

^a^Reaction conditions: 0.8 mL [TBP][4EtOV], 0.5 mol % PdCl_2_(PPh_3_)_2_, 0.75 mmol phenylacetylene, 0.5 mmol iodoaromatic compound, *T* = 55 °C, *t* = 3 h; ^b^isolated yields; ^c^1.25 mmol (2.5 equiv) of phenylacetylene, **3m**: 2,5-bis(2-phenylethynyl)pyridine.

By comparison with the conversion of 4-chloro-1-iodobenzene (**1g**), no formation of 1,4-bis(phenylethynyl)benzene was detected. It agrees with the activated substituents of 2-substituted pyridine derivatives. It can be concluded that by varying electronic and steric properties of substituents of the iodoaromatic substrates at all *ortho*-, *meta*-, and *para*- positions, no significant changes in the product yields were achieved according to previous studies [26,41]. Regarding the negligible influence of the 4-substituents, i.e., no Hammett-plot can be obtained for the above reaction, it can be stated that the rate determining step of the Sonogashira coupling is not related to the formation of Pd-aryl species.

Subsequently, a series of different acetylenes, which can readily be dissolved in [TBP][4EtOV] were subjected to the coupling reaction under identical conditions. By comparison of the efficiency of conversion of iodobenzene and its electron-donating 4-methoxy (**1d**) and electron-withdrawing 4-nitro (**1m**) derivatives with different aliphatic acetylenes (**4**, **6**, and **8**), no significant differences in isolated yields were observed (Table 4, Table 5 and Table 6) verifying the wide range of functional group tolerance by side of acetylenic substrates, as well. Same results were reported by Alper and co-workers, who perform this reaction in [BMIM][PF_6_] as alternative solvent [22].

**Table 4:** Palladium-catalyzed Sonogashira coupling of various iodoaromatic compounds (**1a, d, m**) with propargyl alcohol (**4**).^a^

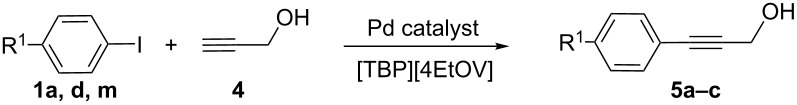				

#		R	Product	Yield (%)^b^

1	**1a**	H	**5a**	80
2	**1m**	NO_2_	**5b**	78
3	**1d**	OCH_3_	**5c**	85

^a^Reaction conditions: 0.8 mL [TBP][4EtOV], 0.5 mol % (PPh_3_)_2_PdCl_2_, 0.75 mmol propargyl alcohol, 0.5 mmol iodoaromatic compounds, *T* = 55 °C, *t* = 3 h; ^b^isolated yields.

**Table 5:** Palladium-catalyzed Sonogashira coupling of various iodoaromatic compounds (**1a, d, m**) with 1-ethynyl-1-cyclohexanol (**6**).^a^

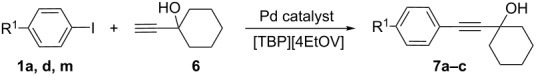				

#		R	Product	Yield (%)^b^

1	**1a**	H	**7a**	85
2	**1m**	NO_2_	**7b**	99
3	**1d**	OCH_3_	**7c**	99

^a^Reaction conditions: 0.8 mL [TBP][4EtOV], 0.5 mol % (PPh_3_)_2_PdCl_2_, 0.75 mmol 1-ethynyl-1-cyclohexanol, 0.5 mmol iodoaromatic compound, *T* = 55 °C, *t* = 3 h; ^b^isolated yields.

**Table 6:** Palladium-catalyzed Sonogashira coupling of various iodoaromatic compounds (**1a, d, m**) with 3-ethyl-1-pentyn-3-ol (**8**).^a^

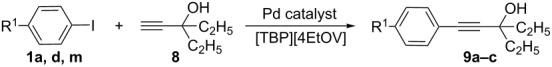				

#		R	Product	Yield (%)^b^

1	**1a**	H	**9a**	87
2	**1m**	NO_2_	**9b**	85
3	**1d**	OCH_3_	**9c**	81

^a^Reaction conditions: 0.8 mL [TBP][4EtOV], 0.5 mol % (PPh_3_)_2_PdCl_2_, 0.75 mmol 3-ethyl-1-pentyn-3-ol, 0.5 mmol iodoaromatic compound, *T* = 55 °C, *t* = 3 h; ^b^isolated yields.

**Figure 2:**
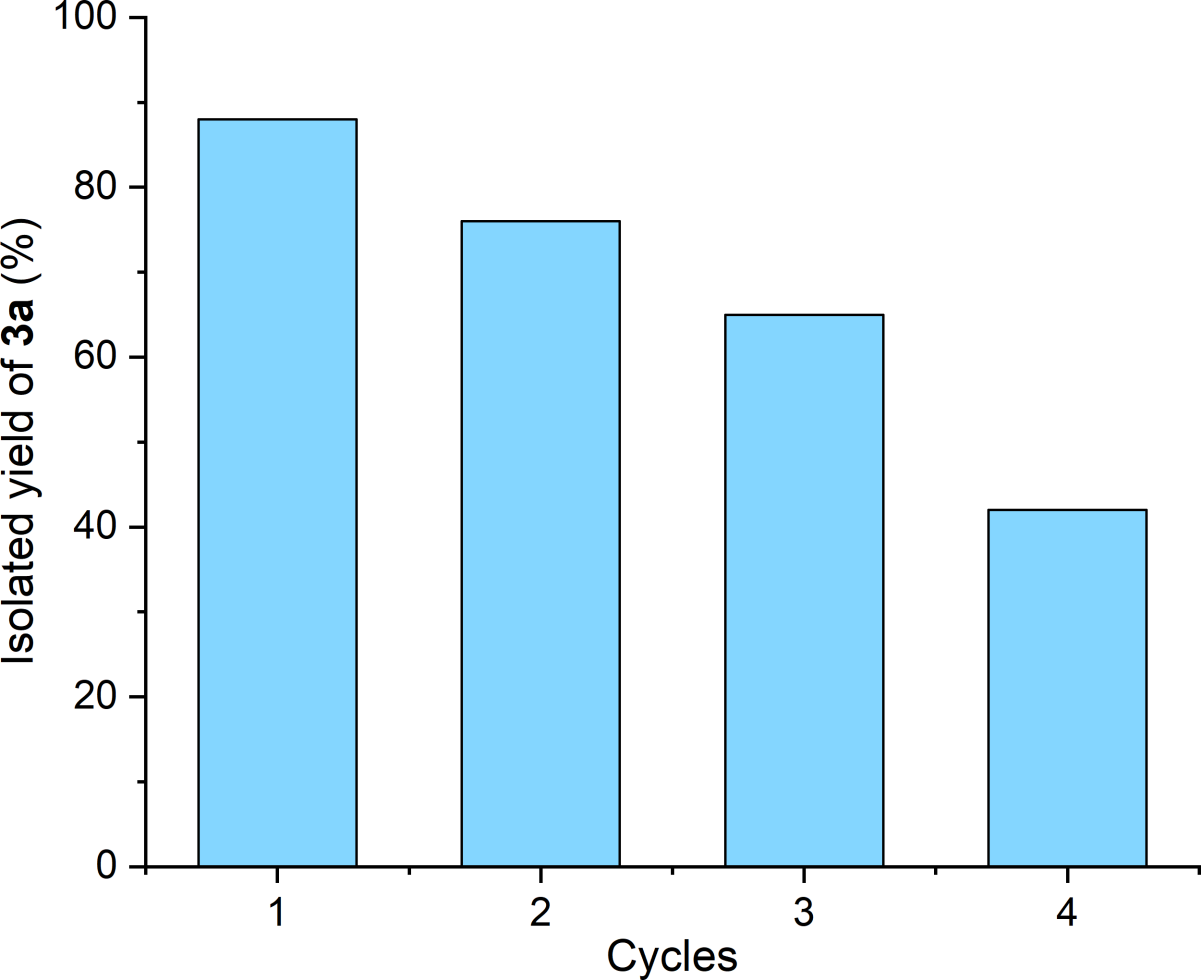
Re-use of Pd catalyst for Sonogashira coupling of iodobenzene (**1a**) and phenylacetylene (**2a**). Reaction conditions: 0.8 mL [TBP][4EtOV], 0.5 mol % catalyst, *T* = 55 °C, *t* = 3 h.

The possible reuse of the catalyst was subsequently investigated by the model reaction of 0.5 mmol of **1a** and 1.5 equiv of **2a** in the presence of 0.5 mol % PdCl_2_(PPh_3_)_2_ at 55 °C for 2 h. After the first extraction of the product from the reaction mixture by the addition of 10 × 5 mL of pentane, **3a** was isolated with a yield of 88%. It should be noted that this reaction verified the reproducibility of the experiments (cf*.* Table 3, entry 1). Same amounts of substrates were added to the Pd catalyst contained in the ionic liquid phase followed by heating to 55 °C. In the second run 12% of decrease in the isolated yield was detected; however, after the 4th cycle, it became significant (Figure 2). The same tendency was reported by Toma for various acetylenes [24]. The ^13^C NMR investigations of the [4EtOV]^−^ anion throughout the reaction did not indicate any change in its composition. Nevertheless, a reaction of the alkoxyvalerate anion with HI that formed during the reaction could be assumed.

## Conclusion

In conclusion, we have demonstrated that a γ-valerolactone-based ionic liquid, tetrabutylphosphonium 4-ethoxyvalerate can be utilized as an alternative solvent for Pd-catalyzed Sonogashira coupling reactions of aryl iodides and functionalized acetylenes under mild conditions. The reactions were performed by using 0.5 mol % catalyst loading and we pointed out that both copper and external base could be eliminated from the reaction mixture without any decrease in catalytic activity. The protocol was tested for a wide range of substrates and several products (**3a**–**n**, **5a–c**, **7a–c**, **9a–c**) were isolated in good to excellent yields. The Cu- and base-free reaction can be performed under air and are highly tolerant to moisture.

## Experimental

The sources of chemicals are listed in Supporting Information File 1. The NMR spectra were recorded on a Brucker Avance 250 MHz spectrometer. The water contents of the ionic liquids were determined by Karl Fischer titration performed by HANNA Instruments 904.

The γ-valerolactone-based ionic liquid ([TBP][4EtOV]) was prepared as described in [34] with details presented in Supporting Information File 1.

Exact mass measurements were performed on a high-resolution Q-Exactive Focus hybrid quadrupole-orbitrap mass spectrometer (Thermo Fisher Scientific, Bremen, Germany) equipped with a heated electrospray ionization (ESI) source. Samples were dissolved in acetonitrile/water 1:1 (v/v) solvent mixture containing 0.1% (v/v) formic acid. Solutions were directly introduced into the ion source using a syringe pump. Under the applied conditions, the compounds form protonated molecules, [M + H]^+^ in positive ionization mode (ESI).

Detailed experimental procedures, characterization data are reported in Supporting Information File 1.

### General procedure for Sonogashira coupling reactions

In a 4 mL screw-cap vial, 0.5 mmol of corresponding iodoarene compound, 1.5 equiv of phenylacetylene or propargyl alcohol, 0.005 equiv PdCl_2_(PPh_3_)_2_, and 0.8 mL of ionic liquid were mixed and stirred at 55 °C for 3 h. After cooling, the mixture was partitioned between 5 mL of water and 5 mL of pentane. After separation, the aqueous phase was extracted subsequently with 2 × 5 mL of pentane. The combined organic phase was washed with brine, dried over MgSO_4_, filtered, and the solvent was evaporated under reduced pressure (ca. 10 mmHg). The oily residue was purified by chromatography on silica gel (Merck Silicagel 60 (0.063−0.200 mm) for column chromatography (70−230 mesh ASTM)) eluted with *n*-pentane/EtOAc. The purity of the isolated products was >98%. The detailed experimental procedure as well as the characterization of isolated compounds are provided in Supporting Information File 1.

## Supporting Information

File 1 Source of chemicals, the detailed experimental procedure as well as characterization data of isolated compounds.

## References

[1] Yang Y, Lan J, You J (2017). Chem Rev.

[2] Shi W, Liu C, Lei A (2011). Chem Soc Rev.

[3] de Meijere A, Diederich F (2004). Metal-Catalyzed Cross-Coupling Reactions.

[4] Johansson Seechurn C C C, Kitching M O, Colacot T J, Snieckus V (2012). Angew Chem, Int Ed.

[5] Devendar P, Qu R-Y, Kang W-M, He B, Yang G-F (2018). J Agric Food Chem.

[6] Jana R, Pathak T P, Sigman M S (2011). Chem Rev.

[7] Chinchilla R, Nájera C (2007). Chem Rev.

[8] Sonogashira K, Negishi E Palladium‐Catalyzed Alkynylation: Sonogashira Alkyne Synthesis. Handbook of Organopalladium Chemistry for Organic Synthesis.

[9] (2019). https://www.fda.gov/media/71737/download.

[10] Severin R, Reimer J, Doye S (2010). J Org Chem.

[11] Karpov A S, Müller T J J (2003). Synthesis.

[12] Panda B, Sarkar T K (2013). Synthesis.

[13] Moon J, Jeong M, Nam H, Ju J, Moon J H, Jung H M, Lee S (2008). Org Lett.

[14] Nagy A, Novák Z, Kotschy A (2005). J Organomet Chem.

[15] Gelman D, Buchwald S L (2003). Angew Chem, Int Ed.

[16] Bakherad M (2013). Appl Organomet Chem.

[17] Markert C, Bannwarth W (2002). Helv Chim Acta.

[18] Akiyama Y, Meng X, Fujita S, Chen Y-C, Lu N, Cheng H, Zhao F, Arai M (2009). J Supercrit Fluids.

[19] Strappaveccia G, Luciani L, Bartollini E, Marrocchi A, Pizzo F, Vaccaro L (2015). Green Chem.

[20] Li J, Yang S, Wu W, Jiang H (2018). Eur J Org Chem.

[21] Lei Z, Chen B, Koo Y-M, MacFarlane D R (2017). Chem Rev.

[22] Bong Park S, Alper H (2004). Chem Commun.

[23] Błaszczyk I, Trzeciak A M, Ziółkowski J J (2009). Catal Lett.

[24] Kmentová I, Gotov B, Gajda V, Toma T (2003). Monatsh Chem.

[25] Fukuyama T, Shinmen M, Nishitani S, Sato M, Ryu I (2002). Org Lett.

[26] Fukuyama T, Rahman M T, Sumino Y, Ryu I (2012). Synlett.

[27] de Lima P G, Antunes O A C (2008). Tetrahedron Lett.

[28] Ermolaev V, Miluykov V, Arkhipova D, Zvereva E, Sinyashin O (2013). Phosphorus, Sulfur Silicon Relat Elem.

[29] Harjani J R, Abraham T J, Gomez A T, Garcia M T, Singer R D, Scammells P J (2010). Green Chem.

[30] Prabhala P, Savanur H M, Kalkhambkar R G, Laali K K (2019). Eur J Org Chem.

[31] Savanur H M, Kalkhambkar R G, Laali K K (2018). Eur J Org Chem.

[32] Iranpoor N, Firouzabadi H, Ahmadi Y (2012). Eur J Org Chem.

[33] Reddy A S, Laali K K (2015). Tetrahedron Lett.

[34] Orha L, Tukacs J M, Gyarmati B, Szilágyi A, Kollár L, Mika L T (2018). ACS Sustainable Chem Eng.

[35] Chinchilla R, Nájera C (2011). Chem Soc Rev.

[36] Hallett J P, Welton T (2011). Chem Rev.

[37] McLachlan F, Mathews C J, Smith P J, Welton T (2003). Organometallics.

[38] Siemsen P, Livingston R C, Diederich F (2000). Angew Chem, Int Ed.

[39] Li J-H, Liang Y, Zhang X-D (2005). Tetrahedron.

[40] (2019). Triethylamine.

[41] Liang Y, Xie Y-X, Li J-H (2006). J Org Chem.

