# Supplementary material for: Palladium-catalyzed Sonogashira coupling reactions in γ-valerolactone-based ionic liquids
Source: Beilstein J Org Chem. 2019 Dec 3;15:2907–13. doi: 10.3762/bjoc.15.284 (PMC6902853; doi:10.3762/bjoc.15.284)
Supplement: File 1 — Source of chemicals, the detailed experimental procedure as well as characterization data of isolated compounds. [file Beilstein_J_Org_Chem-15-2907-s001.pdf]

BEILSTEIN JOURNAL OF ORGANIC CHEMISTRY

Supporting Information

for

Palladium-catalyzed Sonogashira coupling reactions in γ-valerolactone-based ionic liquids

László Orha, József M. Tukacs, László Kollár and László T. Mika

Beilstein J. Org. Chem. **2019,** *15,* 2907–2913. doi:10.3762/bjoc.15.284

Source of chemicals, the detailed experimental procedure as well as characterization data of isolated compounds

License and Terms: This is a supporting information file under the terms of the Creative Commons Attribution License (http://creativecommons.org/licenses/by/4.0). Please note that the reuse, redistribution and reproduction in particular requires that the authors and source are credited.
The license is subject to the *Beilstein Journal of Organic Chemistry* terms and conditions: (https://www.beilstein-journals.org/bjoc)

Source of chemicals

γ-Valerolactone, phenylacetylene, propargyl alcohol, 1-ethynyl-1-cyclohexanol, 3-ethyl-1-pentyn-3-ol, iodobenzene and its substituted derivatives, copper iodide, 1-butyl-3-methylimidazolium tetrafluoroborate, 1-butyl-3-methylimidazolium hexafluorophoshate, 1-butyl-3-methylimidazolium octylsulfate, 1-ethyl-3-methylimidazolium tetrafluoroborate, triethylamine, and palladium-catalyst precursors were purchased from Sigma-Aldrich Kft., Budapest, Hungary and used as received. Solvents were obtained from Molar Chemicals Ltd., Budapest, Hungary and used without further purification.

Preparation of tetrabutylphosphonium 4-ethoxyvalerate ([TBP][4EtOV])

A mixture of 8.71 g (50 mmol) of ethyl 4-ethoxyvalerate and a 40% aqueous solution of 13.82 g (50 mmol) of tetrabutylphosphonium hydroxide was stirred in 10 ml water at 60 °C for 1 h, during which time the two-phase system turned into a homogeneous solution. The water formed was removed under reduced pressure, then 5 × 5 ml hexane was distilled from the oily residue under reduced pressure, then was dried at 80 °C in vacuum (0.5 mmHg). Yield: 20,01 g (99%) as a light-yellow solid. ^{1}H-NMR, ^{13}C-NMR and ^{31}P-NMR correspond to our published results.[1]

Preparation and characterization of acetylenes presented in Table 3

Diphenylacetylene (3a)

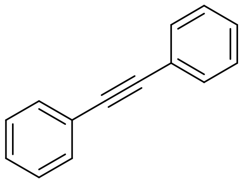

The general procedure was followed using 56 µl (0.5 mmol) iodobenzene, 82 µl (0.75 mmol) phenylacetylene, 1.8 mg (0.025 mmol) $(PPh_3)_2PdCl_2$, and 0.8 ml of [TBP][4EtOV] ionic liquid as solvent. Yield: 75.7 mg (85%) as white solid. ^{1}H NMR (250 MHz, $CDCl_3$): δ (ppm) 7.21–7.33 (m, 6H); 7.38–7.52 (m, 4H). It corresponds to the published results.[2]

1-Methyl-2-phenylethynylbenzene (3b)

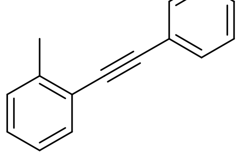

The general procedure was followed using 64 µl (0.5 mmol) 2-iodotoluene, 82 µl (0.75 mmol) phenylacetylene, 1.8 mg (0.025 mmol) $(PPh_3)_2PdCl_2$, and 0.8 ml of [TBP][4EtOV] ionic liquid as solvent. Yield: 92.9 mg (96%) as colorless oil. ^{1}H NMR (250 MHz, $CDCl_3$): δ (ppm) 2.55 (s, 3H), 7.14–7.23 (m, 1H), 7.23-7.29 (d, 2H), 7.33–7.41 (m, 3H), 7.50–7.61 (m, 3H). It corresponds to the published results.[3]

1-Methyl-4-phenylethynylbenzene (3c)

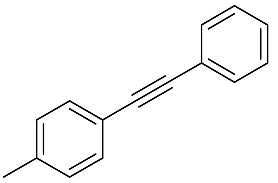

The general procedure was followed using 109.0 mg (0.5 mmol) 4-iodotoluene, 82 µl (0.75 mmol) phenylacetylene, 1.8 mg (0.025 mmol) $(PPh_3)_2PdCl_2$, and 0.8 ml of [TBP][4EtOV] ionic liquid as solvent. Yield: 91.4 mg (95%) as white solid. 1H NMR (250 MHz, $CDCl_3$): δ (ppm) 2.38 (s, 3H), 7.17 (d, 2H), 7.30–7.38 (m, 3H), 7.44 (d, 2H), 7.49–7.57 (m, 2H). It corresponds to the published results.[4]

1-Methoxy-4-phenylethynylbenzene (3d)

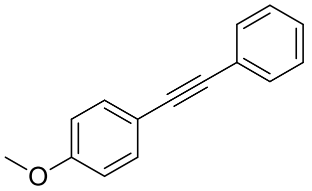

The general procedure was followed using 117.0 mg (0.5 mmol) 4-iodoanisole, 82 µl (0.75 mmol) phenylacetylene, 1.8 mg (0.025 mmol) $(PPh_3)_2PdCl_2$, and 0.8 ml of [TBP][4EtOV] ionic liquid as solvent. Yield: 85.4 mg (82%) as white solid. ^1H NMR (250 MHz, $CDCl_3$): δ (ppm) 3.66 (s, 1H), 6.74 (d, 2H), 7.15–7.24 (m, 3H), 7.36 (d, 2H), 7.38–7.44 (m, 2H). It corresponds to the published results.[5]

1-Fluoro-4-phenylethynylbenzene (3e)

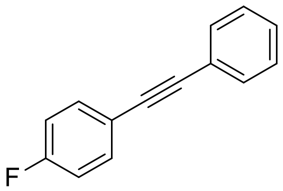

The general procedure was followed using 58 µl (0.5 mmol) 1-iodo-4-fluorobenzene, 82 µl (0.75 mmol) phenylacetylene, 1.8 mg (0.025 mmol) $(PPh_3)_2PdCl_2$, and 0.8 ml of [TBP][4EtOV] ionic liquid as solvent. Yield: 78.0 mg (80%) as colorless oil. ^1H NMR (250 MHz, $CDCl_3$): δ (ppm) 7.05 (t, 2H), 7.31–7.39 (m, 3H), 7.46, 7.59 (m, 4H). It corresponds to the published results.[6]

1-Chloro-4-phenylethynylbenzene (3f)

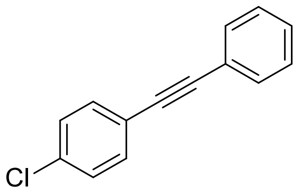

The general procedure was followed using 119.2 mg (0.5 mmol) 1-chloro-4-iodoobenzene, 82 µl (0.75 mmol) phenylacetylene, 1.8 mg (0.025 mmol) $(PPh_3)_2PdCl_2$, and 0.8 ml of [TBP][4EtOV] ionic liquid as solvent. Yield: 92.1 mg (87%) as colorless oil. ^1H NMR (250 MHz, $CDCl_3$): δ (ppm) 7.39–7.49 (m, 5H), 7.47 (d, 2H), 7.50–7.57 (m, 2H). It corresponds to the published results.[16]

1-Bromo-4-phenylethynylbenzene (3g)

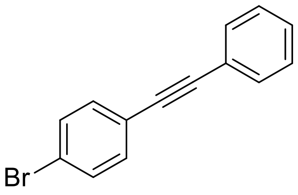

The general procedure was followed using 141.5 mg (0.5 mmol) 1-bromo-4-iodobenzene, 82 µl (0.75 mmol) phenylacetylene, 1.8 mg (0.025 mmol) $(PPh_3)_2PdCl_2$, and 0.8 ml of [TBP][4EtOV] ionic liquid as solvent. Yield: 66.8 mg (52%) as pale-yellow solid. ^1H NMR (250 MHz, $CDCl_3$): δ (ppm) 7.30–7.39 (m, 3H), 7.39 (d, 2H), 7.49 (d, 2H), 7.51–7.58 (m, 2H). It corresponds to the published results.[7]

2-(2-Phenylethynyl)thiophene (3h)

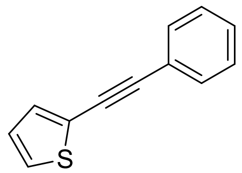

The general procedure was followed using 55 µl (0.5 mmol) 2-iodothiophene, 82 µl (0.75 mmol) phenylacetylene, 1.8 mg (0.025 mmol) $(PPh_3)_2PdCl_2$, and 0.8 ml of [TBP][4EtOV] ionic liquid as solvent. Yield: 74.2 mg (80%) as colorless oil. ^1H NMR (250 MHz, $CDCl_3$): δ (ppm) 7.03 (m, 1H), 7.28–7.33 (m, 2H), 7.35–7.39 (m, 3H), 7.50–7.59 (m, 2H). It corresponds to the published results.[8]

3-(2-Phenylethynyl)pyridine (3i)

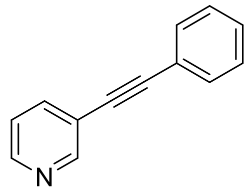

The general procedure was followed using 102.5 mg (0.5 mmol) 3-iodopyridine, 82 µl (0.75 mmol) phenylacetylene, 1.8 mg (0.025 mmol) $(PPh_3)_2PdCl_2$, and 0.8 ml of [TBP][4EtOV] ionic liquid as solvent. Yield: 67.3 mg (75%) as colorless oil. ^1H NMR (250 MHz, $CDCl_3$): δ (ppm) 7.22–7.31 (m, 1H), 7.31–7.41 (m, 3H), 7.48–7.61 (m, 2H), 7.80 (td, 1H), 8.54 (d, 1H), 8.77 (s, 1H). It corresponds to the published results.[9]

2-Amino-3-(2-phenylethynyl)pyridine (3j)

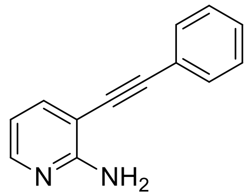

The general procedure was followed using 110.0 mg (0.5 mmol) 2-amino-3-iodopyridine, 82 µl (0.75 mmol) phenylacetylene, 1.8 mg (0.025 mmol) $(PPh_3)_2PdCl_2$, and 0.8 ml of [TBP][4EtOV] ionic liquid as solvent. Yield: 90.5 mg (93%) as light-yellow solid. ^1H NMR (250 MHz, $CDCl_3$): δ (ppm) 6.55–6.73 (m, 1H), 7.20–7.45 (m, 3H), 7.46–7.76 (m, 3H), 7.99–8.15 (m, 1H), 5.21 (br.s, 2H). It corresponds to the published results.[10]

2-Chloro-1-(2-phenylethynyl)-4-trifluoromethylbenzene (3k)

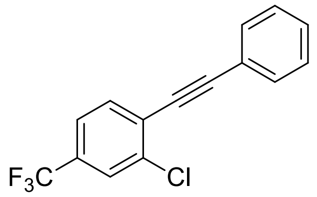

The general procedure was followed using 77 µl (0.5 mmol) 3-chloro-4-iodobenzotrifluoride, 82 µl (0.75 mmol) phenylacetylene, 1.8 mg (0.025 mmol) $(PPh_3)_2PdCl_2$, and 0.8 ml of [TBP][4EtOV] ionic liquid as solvent. Yield: 110.6 mg (79%) as colorless oil. ^1H NMR (250 MHz, $CDCl_3$): δ (ppm) 7.29-7.42 (m, 3H), 7.43-7.51 (m, 1H), 7.51-7.61 (m, 2H), 7.61-7.72 (m, 2H). ^{13}C NMR (62.8 MHz, $CDCl_3$), δ (ppm) 85.4, 97.5, 122.7, 123.5 (q, J = 272 Hz, CF_3), 123.7 (q, J = 3.6 Hz), 126.7 (q, J = 3.8 Hz), 127.4, 128.9, 129.6, 131.4 (q, J = 33 Hz), 132.3, 133.8, 136.9. HRMS: Calculated: 281.0339, Measured: 281.03377 (-0.60 ppm)

2-Chloro-5-(2-phenylethynyl)pyridine (3l)

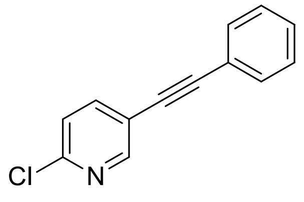

The general procedure was followed using 119.7 mg (0.5 mmol) 2-chloro-5-iodopyridine, 55 µl (0.5 mmol) phenylacetylene, 1.8 mg (0.025 mmol) $(PPh_3)_2PdCl_2$, and 0.8 ml of [TBP][4EtOV] ionic liquid as solvent. Yield: 76.9 mg (72%) as light-yellow solid. ^1H NMR (250 MHz, $CDCl_3$): δ (ppm) 7.21 (d,

1H), 7.24–7.34 (m, 3H), 7.38–7.49 (m, 2H), 7.65 (d, 1H), 8.44 (s, 1H). ^{13}C NMR (62.8 MHz, $CDCl_3$), δ (ppm) 85.1, 94.2, 119.8, 122.5, 124.2, 128.9, 129.4, 132.1, 141.2, 150.8, 152.4. HRMS: Calculated: 214.0418, Measured: 214.04164 (–0.76 ppm)

2,5-Bis(2-phenylethynyl)pyridine (3m)

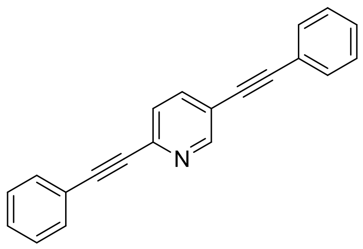

The general procedure was followed using 119.7 mg (0.5 mmol) 2-chloro-5-iodopyridine, 137 µl (1.25 mmol, 2.5 equiv) phenylacetylene, 1.8 mg (0.025 mmol) $(PPh_3)_2PdCl_2$, and 0.8 ml of [TBP][4EtOV] ionic liquid as solvent. Yield: 96.3 mg (69%) as light-yellow solid. ^1H NMR (250 MHz, $CDCl_3$): δ (ppm) 7.22-7.34 (m, 6H), 7.41 (d, 1H), 7.44-7.55 (m, 4H), 7.69 (d, 1H), 8.67 (s, 1H). It corresponds to the published results.[11]

Preparation and characterization of acetylenes presented in Table 4

3-Phenylprop-2-yn-1-ol (5a)

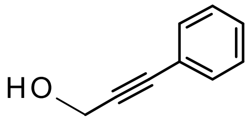

The general procedure was followed using 56 µl (0.5 mmol) iodobenzene, 44 µl (0.75 mmol) propargyl alcohol, 1.8 mg (0.025 mmol) $(PPh_3)_2PdCl_2$, and 0.8 ml of [TBP][4EtOV] ionic liquid as solvent. Yield: 53.4 mg (80%) as light-yellow oil. ^1H NMR (250 MHz, $CDCl_3$): δ (ppm) 4.49 (s, 2H), 7.33–7.25 (m, 3H), 7.47–7.38 (m, 2H). It corresponds to the published results.[12]

3-(4-Nitrophenyl)prop-2-yn-1-ol (5b)

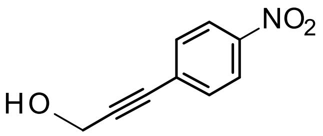

The general procedure was followed using 124.5 mg (0.5 mmol) 1-iodo-4-nitrobenzene, 44 µl (0.75 mmol) propargyl alcohol, 1.8 mg (0.025 mmol) $(PPh_3)_2PdCl_2$, and 0.8 ml of [TBP][4EtOV] ionic liquid as solvent. Yield: 86.6 mg (78%) as light-yellow solid. ^1H NMR (250 MHz, $CDCl_3$): δ (ppm) 4.53 (s, 2H), 7.56 (d, 2H), 8.18 (d, 2H). It corresponds to the published results.[13]

3-(4-Methoxyphenyl)prop-2-yn-1-ol (5c)

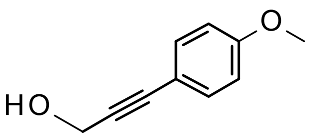

The general procedure was followed using 117.0 mg (0.5 mmol) 4-iodo-anisole, 44 µl (0.75 mmol) propargyl alcohol, 1.8 mg (0.025 mmol) $(PPh_3)_2PdCl_2$, and 0.8 ml of [TBP][4EtOV] ionic liquid as solvent. Yield: 69.2 mg (85%) as light-yellow oil. ^1H NMR (250 MHz, $CDCl_3$): δ (ppm) 3.79 (s, 3H), 4.46 (s, 2H), 6.82 (d, 2H), 7.35 (d, 2H). It corresponds to the published results.[14]

Preparation and characterization of acetylenes presented in Table 5

1-(2-Phenylethynyl)cyclohexanol (7a)

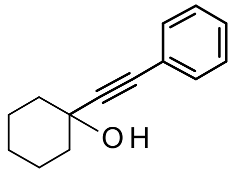

The general procedure was followed using 56 μl (0.5 mmol) iodobenzene, 93.1 mg (0.75 mmol) 1-ethynyl-1-cyclohexanol, 1.8 mg (0.025 mmol) $(PPh_3)_2PdCl_2$, and 0.8 ml of [TBP][4EtOV] ionic liquid as solvent. Yield: 85.7 mg (85%) as light-yellow solid. ^1H NMR (250 MHz, $CDCl_3$): δ (ppm) 1.17–1.34 (m, 1H), 1.49–1.84 (m, 8H), 1.88–2.08 (m, 2H), 2.16 (s, 1H), 7.21-7.36 (m, 3H), 7.38–7.48 (m, 2H). It corresponds to the published results.[15]

1-[2-(4-Nitrophenyl)ethynyl]cyclohexanol (7b)

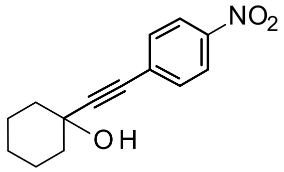

The general procedure was followed using 124.5 mg (0.5 mmol) 1-iodo-4-nitrobenzene, 93.1 mg (0.75 mmol) 1-ethynyl-1-cyclohexanol, 1.8 mg (0.025 mmol) $(PPh_3)_2PdCl_2$, and 0.8 ml of [TBP][4EtOV] ionic liquid as solvent. Yield: 122 mg (99%) as light-yellow solid. ^1H NMR (250 MHz, $CDCl_3$): δ (ppm) 1.22–1.41 (m, 1H), 1.45–1.86 (m, 8H), 1.94–2.10 (m, 2H), 7.55 (d, 2H), 8.17 (d, 2H). It corresponds to the published results.[16]

1-[2-(4-Methoxyphenyl)ethynyl]cyclohexanol (7c)

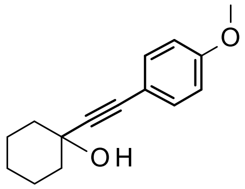

The general procedure was followed using 56 μl (0.5 mmol) iodobenzene, 93.1 mg (0.75 mmol) 1-ethynyl-1-cyclohexanol, 1.8 mg (0.025 mmol) $(PPh_3)_2PdCl_2$, and 0.8 ml of [TBP][4EtOV] ionic liquid as solvent. Yield: 115 mg (99%) as light-yellow solid. ^1H NMR (250 MHz, $CDCl_3$): δ (ppm) 1.18–1.34 (m, 1H), 1.48–1.81 (m, 8H), 1.85–2.12 (m, 3H), 3.81 (s, 3H), 6.82, (d, 2H), 7.36 (d, 2H). It corresponds to the published results.[17]

Preparation and characterization of acetylenes presented in Table 6

3-Ethyl-1-phenyl-pent-1-yn-3-ol (9a)

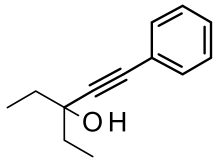

The general procedure was followed using 56 μl (0.5 mmol) iodobenzene, 96 μl (0.75 mmol) 3-ethyl-1-pentyn-3-ol, 1.8 mg (0.025 mmol) $(PPh_3)_2PdCl_2$, and 0.8 ml of [TBP][4EtOV] ionic liquid as solvent. Yield: 76.9 mg (87%) as light-yellow oil. ^1H NMR (250 MHz, $CDCl_3$): δ (ppm) 1.10 (t, 6H), 1.69–1.82 (m, 4H), 2.03 (s, 1H), 7.27–7.33 (m, 3H), 7.37–7.45 (m, 2H). It corresponds to the published results.[18]

3-Ethyl-1-(4-nitrophenyl)-pent-1-yn-3-ol (9b)

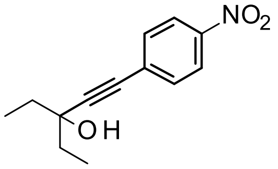

The general procedure was followed using 124.5 mg (0.5 mmol) 1-iodo-4-nitrobenzene, 96 µl (0.75 mmol) 3-ethyl-1-pentyn-3-ol, 1.8 mg (0.025 mmol) $(PPh_3)_2PdCl_2$, and 0.8 ml of [TBP][4EtOV] ionic liquid as solvent. Yield: 99.1 mg (85%) as light yellow oil. ^{1}H NMR (250 MHz, $CDCl_3$): δ (ppm) 1.10 (t, 6H), 1.72–1.86 (m, 4H), 2.04 (s, 1H), 7.56 (d, 2H), 8.17 (d, 2H). It corresponds to the published results.[19]

3-Ethyl-1-(4-methoxyphenyl)-pent-1-yn-3-ol (9c)

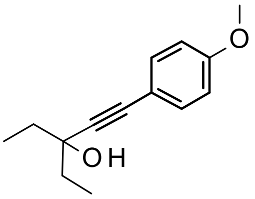

The general procedure was followed using 117.0 mg (0.5 mmol) 4-iodoanisole, 96 µl (0.75 mmol) 3-ethyl-1-pentyn-3-ol, 1.8 mg (0.025 mmol) $(PPh_3)_2PdCl_2$, and 0.8 ml of [TBP][4EtOV] ionic liquid as solvent. Yield: 88.7 mg (81%) as light-yellow oil. ^{1}H NMR (250 MHz, $CDCl_3$): δ (ppm) 1.09 (t, 6H), 1.69–1.81 (m, 4H), 1.99 (s, 1H), 3.80 (s, 3H), 6.83 (d, 2H), 7.35 (d, 2H). ^{13}C NMR (62.9 MHz, $CDCl_3$): δ (ppm) 9.0, 34.8, 55.6, 72.8, 84.8, 90.6, 114.6, 115.4, 1335, 159.8. HRMS Calculated: 219.1380, Measured: 219.13761 (–1.58 ppm)

References

1 L. Orha, J. M. Tukacs, B. Gyarmati, A. Szilágyi, L. Kollár, L. T. Mika, *ACS Sustainable Chem. Eng*. **2018**, 6, 5097.

2 Wardrop, Duncan J.; Komenda, John P. *Org. Lett*. **2012,** 14, 1548.

3 Shi, Leilei; Jia, Wei; Li, Xun; Jiao, Ning *Tetrahedron Lett*. **2013**, 54, 1951.

4 Buxaderas, Eduardo; Alonso, Diego A.; Najera, Carmen *Eur. J. Org. Chem*. **2013**, 26, 5864.

5 Wardrop, Duncan J.; Komenda, John P. *Org. Lett*., **2012**, 14, 1548.

6 Li, Tingyi; Sun, Peng; Yang, Hailong; Zhu, Yan; Yan, Hong; Lu, Linhua; Mao, Jincheng *Tetrahedron* **2012,** 68, 6413.

7 Xu, Cheng-Fu; Xu, Mei; Jia, Yi-Xia; Li, Chuan-Ying *Org. Lett*. **2011**. 13, 1556.

8 Garcia Ruano, Jose Luis; Aleman, Jose; Marzo, Leyre; Alvarado, Cuauhtemoc; Tortosa, Mariola; Diaz-Tendero, Sergio; Fraile, Alberto *Chem. Eur. J*. **2012,** 18, 8414.

9 Li, Xi'Ang; Yang, Fan; Wu, Yangjie *J. Org. Chem*. **2013**, 78, 4543.

10 De Mattos, Marcos Carlos; Alatorre-Santamaria, Sergio; Gotor-Fernandez, Vicente; Gotor, Vicente *Synthesis* **2007,** 2149.

11 Kang, Dongjin; Eom, Dahan; Kim, Hyunseok; Lee, Phil Ho Eur. *J. Org. Chem*. **2010**, 2330.

12 Wang, Lin; Sun, Hongjian; Li, Xiaoyan *Eur. J. Inorg. Chem*. **2015,** 2732.

13 Nowak-Krol, Agnieszka; Koszarna, Beata; Yoo, Su Yeon; Chrominski, Jan; Weclawski, Marek K.; Lee, Chang-Hee; Gryko, Daniel T. *J. Org. Chem*. **2011,** 76, 2627.

14 Dell'Isola, Antonio; McLachlan, Matthew M. W.; Neuman, Benjamin W.; Al-Mullah, Hawaa M. N.; Binks, Alexander W. D.; Elvidge, Warren; Shankland, Kenneth; Cobb, Alexander J. A. *Chem. Eur. J.* **2014**, 20, 11685.

15 Belov, Dmitry S.; Lukyanenko, Evgeny R.; Kurkin, Alexander V.; Yurovskaya, Marina A. J. *Org. Chem.*, **2012**, 77, 10125.

16 Gholap, Atul R.; Venkatesan; Pasricha, Renu; Daniel, Thomas; Lahoti, Rajgopal J.; Srinivasan, Kumar V. *J. Org. Chem*. **2005**, 70, 4869.

17 Hussain, Mohd. Kamil; Ansari, Mohd. Imran; Kant, Ruchir; Hajela, Kanchan *Org. Lett*., **2014**, 16, 560.

18 Ying, Jun; Zhou, Chao; Wu, Xiao-Feng *Org. Biomol. Chem*. **2018**, 16, 1065.

19 Olaizola, Iurre; Campano, Teresa E.; Iriarte, Igor; Vera, Silvia; Mielgo, Antonia; García, Jesús M.; Odriozola, José M.; Oiarbide, Mikel; Palomo, Claudio *Chem. Eur. J*., **2018**, 24, 3893.
